# Influence of 17β-Estradiol on Gene Expression of *Paracoccidioides* during Mycelia-to-Yeast Transition

**DOI:** 10.1371/journal.pone.0028402

**Published:** 2011-12-14

**Authors:** Jata Shankar, Thomas D. Wu, Karl V. Clemons, Jomar P. Monteiro, Laurence F. Mirels, David A. Stevens

**Affiliations:** 1 California Institute for Medical Research, Santa Clara Valley Medical Center, San Jose, California, United States of America; 2 Division of Infectious Diseases, Department of Medicine, Santa Clara Valley Medical Center, San Jose, California, United States of America; 3 Division of Infectious Diseases and Geographic Medicine, Department of Medicine, Stanford University, Stanford, California, United States of America; 4 Department of Bioinformatics and Computational Biology, Genentech, Inc., South San Francisco, California, United States of America; Université de Nice-CNRS, France

## Abstract

**Background:**

*Paracoccidioides* is the causative agent of paracoccidioidomycosis, a systemic mycosis endemic to Latin America. Infection is initiated by inhalation of conidia (C) or mycelial (M) fragments, which subsequently differentiate into yeast (Y). Epidemiological studies show a striking predominance of paracoccidioidomycosis in adult men compared to premenopausal women. *In vitro* and *in vivo* studies suggest that the female hormone (17β-estradiol, E_2_) regulates or inhibits M-or-C-to-Y transition. In this study we have profiled transcript expression to understand the molecular mechanism of how E_2_ inhibits M-to-Y transition.

**Methodology:**

We assessed temporal gene expression in strain Pb01 in the presence or absence of E_2_ at various time points through 9 days of the M-to-Y transition using an 11,000 element random-shear genomic DNA microarray and verified the results using quantitative real time-PCR. E_2_-regulated clones were sequenced to identify genes and biological function.

**Principal Findings:**

E_2_-treatment affected gene expression of 550 array elements, with 331 showing up-regulation and 219 showing down-regulation at one or more time points (p≤0.001). Genes with low expression after 4 or 12 h exposure to E_2_ belonged to pathways involved in heat shock response (*hsp90* and *hsp70*), energy metabolism, and several retrotransposable elements. Y-related genes, α-1,3-glucan synthase, mannosyltransferase and *Y20*, demonstrated low or delayed expression in E_2_-treated cultures. Genes potentially involved in signaling, such as palmitoyltransferase (erf2), small GTPase RhoA, phosphatidylinositol-4-kinase, and protein kinase (serine/threonine) showed low expression in the presence of E_2_, whereas a gene encoding for an arrestin domain-containing protein showed high expression. Genes related to ubiquitin-mediated protein degradation, and oxidative stress response genes were up-regulated by E_2_.

**Conclusion:**

This study characterizes the effect of E_2_ at the molecular level on the inhibition of the M-to-Y transition and is indicative that the inhibitory actions of E_2_ may be working through signaling genes that regulate dimorphism.

## Introduction

*Paracoccidioides* is a thermally dimorphic fungus that is the etiological agent of paracoccidioidomycosis (PCM) [1]. Until recently, *P. brasiliensis* has been considered the only species in this genus. However, assessment of the molecular, phylogenetic analysis, and morphological characteristics of numerous isolates of *Paracoccidioides* has resulted in the differentiation of the genus into two species, *P. brasiliensis* and *P. lutzii*, the latter recently proposed as the new species comprised of the Pb01-like isolates [2]. PCM, endemic to Central and South America, is one of the most common systemic mycoses in these areas with a striking predominance of clinical disease in adult men [3], [4]. Infection usually occurs after inhalation of the saprophytic form conidia (C) or mycelial (M) propagules, which reach the alveolar epithelium, where they transform into the parasitic yeast (Y) form. The temperature of 37°C triggers its dimorphism in the host or in culture, which results in changes in cellular and cell wall constituents [5], [6]. Genomic approaches, such as EST (expressed sequence tag) analysis or suppression subtraction hybridization, and cDNA or gDNA microarrays have been used to identify differentially expressed genes in each morphological phase, during M-or-C-to-Y transition, or during host-pathogen interaction [7]–[19]. These studies have highlighted how this fungus adapts to its environment.

The morphological differentiation that takes place during M-or-C-to-Y transition is considered vital in the pathogenesis of PCM; strains that are unable to transform into yeasts are not virulent [20], [21]. Epidemiological studies of PCM patients indicate that the disease is about 11 to 30 times more common in adult males despite equal frequencies of exposure to this fungus by both genders [4], [22]–[28]. Furthermore, clinical disease in prepubertal or postmenopausal females is equal to that in males [4]. This epidemiological feature of PCM led to the hypothesis of a steroidal interaction between mammalian hormones and this organism. *In vitro* studies have shown that E_2_ inhibits the M-or-C-to-Y transition [29], [30]. Additional studies have shown high affinity stereo-specific binding of E_2_ to a cytosolic protein from mycelia or yeast [31], [32]. Furthermore, blocking of dimorphism was demonstrated in animal studies [33], [34]. Since many fungi interact with pheromones or hormones, which modulate fungal behavior [35], fungal genes responsive to hormones are of great interest and possess potential clinical relevance. Thus, our aim in this study was to examine the influence of E_2_ on gene expression by *Paracoccidioides* during thermally induced morphological transition from the M to the Y-form, and determine which, if any, genes might show an altered expression in the presence of E_2_. Our results indicate changes in the expression of various genes in different functional categories.

## Results

The goal of our study was to delineate the effects of E_2_ on *Paracoccidioides* while the organism was undergoing its thermally-induced form transition from the infective M-form to the Y-form found in infected tissues. We approached this goal by first examining the morphologic transition to confirm that E_2_ was inhibiting it, and then sampling the fungal mass from those same cultures to develop a transcriptional profile of the organism during form transition.

### E_2_ effects

#### Effects on M-to-Y transition

**Figure 1:**
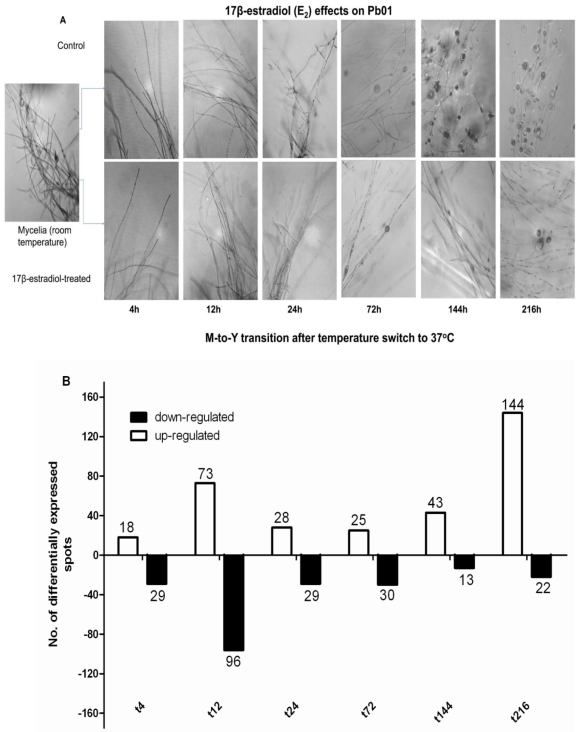
Temperature induced mycelia-to-yeast transition in Pb01. Temperature induced mycelia-to-yeast transition in Pb01. at various time-points in control and E_2_-treated samples, ×400 magnification (1A). Number of array elements showing significant (p≤0.001) differential expression in E_2_-treated samples in comparison to control at various times during morphologic transition (1B).

Following the temperature switch of a mycelia culture from 25°C to 37°C, hyphae were maintained morphologically through the 12 h time point, and were indistinguishable among controls or E_2_-treated cultures. At the 24 h time point, prominent changes in the hyphal morphology, characterized by intercalary or lateral swellings, were observed in controls, but not the E_2_-treated cultures. At the 72 h time point, we observed more transforming hyphae in the controls than in E_2_-treated samples. Transformation into yeast, characterized by protrusion of buds or multiple buds from chlamydospores, was observed at 144 h. Free mature, multiply budding yeast were observed after 216 h in the control samples. In contrast, hyphae were maintained in E_2_-treated samples until the 144 h and 216 h time points, with only rare cells in hyphae showing a rounding or progression through transition into a Y-form (Figure 1A). These data are indicative that Pb01 responds functionally to the presence of E_2_ (i.e., E_2_ inhibition of M-to-Y transition) in the same manner as did *P. brasiliensis* in our previous studies [29], [36], [37]. This validates our use of the Pb01 strain for the current studies.

#### E_2_ effects on transcriptional profile

We assessed gene expression of Pb01 during M-to-Y transition at various times through 216 h of transition by evaluating transcripts for changes in expression in the presence or absence of E_2_. We employed a random-shear genomic DNA microarray containing over 11,000 elements, previously developed in our laboratory [8]. In comparison with controls, E_2_ treatment significantly altered transcript levels (using a p-value cutoff of 1e-3) in genes represented by 550 array elements, with 331 of the elements showing upregulation and 219 showing down-regulation at one or more time points (Figure 1B).

**Figure 2:**
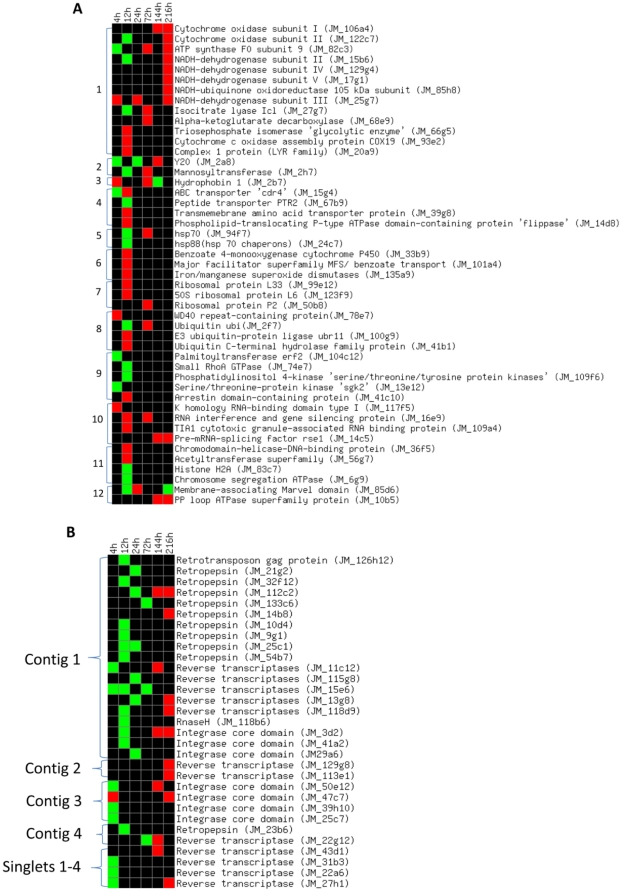
E_2_ regulated Pb01 genes during M-to-Y transition. In comparison to controls, E_2_ regulated Pb01 genes assigned to a functional category that have significant change of expression values at selected time points during M-to-Y transition. 1; Energy production, 2; Yeast genes, 3; Mycelial gene, 4; Transporters, 5; Heat-shock response, 6; Oxidative stress, 7; Ribosomal proteins, 8; Ubiquitin-mediated protein degradation, 9; Signal transduction, 10; RNA processing, 11; Chromatin structure, 12; Others (2A). E_2_ regulated retrotransposable elements; 31 sequenced clones with sequence similarity to retrotransposon elements (2B). Red represents significantly higher expression and green represents a significantly low level of expression, from microarray data. Genes with a p≥0.001 were set to black at all time points. The heat-map was prepared using online software available at http://www.bioinformatics.ubc.ca.

We felt that gene expression during the earlier stages of M-to-Y transition might be the most important in regulating the change in morphology. Thus, we sequenced and annotated 92 array elements showing significantly altered transcript levels at earlier time points (i.e., 4, 12, or 24 h) in E_2_-treated in comparison to controls. In addition, we searched the previously sequenced array elements [8] for changes in transcript expression and compiled the results. From 816 sequenced array elements, 164 showed altered mRNA expression in the presence of E_2_ at one or more time points. However, 21 and 29 sequenced elements showed non-specific and no hits in the database, respectively, and were excluded from the analysis. Subsequent analysis showed that 47 array elements could be assigned to a functional category. These are listed, with their expression values, in Table 1 and 2; 33 elements showing sequence similarity only with hypothetical or predicted proteins and 3 with ESTs of Pb01 with unknown function are excluded from the table. The annotated function of the 47 genes included a variety of categories, such as energy metabolism, cell structure and morphology, transporters, heat-shock response, oxidative stress response, ribosomal proteins, ubiquitin-mediated protein degradation, signal transduction, RNA processing, chromatin structure, and others. We also found 31 array elements that had sequence similarity with retrotransposon elements. A heat map of temporal transcript expression of 47 functional category genes and 31 retrotransposon array elements in the presence of E_2_ in comparison to the transcript expression of the pooled controls during form-transition is presented in figures 2A and 2B, respectively.

**Table 1:** Sequence analysis of E_2_ regulated gene and expression values in comparison to controls at various time-points during M-to-Y transition.

Gene product		Accession number (GI)	BLASTX (Fungi); E-value	M	E_2_:Control (Ratio)					
				Hours	Hours					
				0	4	12	24	72	144	216
**1**	Isocitrate lyase ‘icl’ (JM_27g7)	226280967	3e-34	2.85	0.06	0.09	0.76	5.21	0.47	0.85
	*ATP synthase F0 subunit-9 (JM_82c3)	68302975	2e-12	0.005	0.09	0.26	1.53	4.42	4.64	12.1
	*Cytochrome oxidase subunit I (JM_106a4)	68302975	4e-72	0.0007	1.44	0.39	3.87	1.43	5.89	16.9
	*Cytochrome oxidase subunit II (JM_122c7)	68302975	1e-61	0.012	0.6	0.009	2.84	3.48	4.17	14.2
	Cytochrome c oxidase assembly protein ‘cox19’ (JM_93e2)	226285269	6e-23	1.49	0.74	6.71	0.89	1.63	1.16	0.57
	NADH-dehydrogenasesubunit III (JM_25g7)	63081165	8e-006	0.0003	2.28	0.18	3.35	2.01	2.34	12.6
	Complex 1 protein (LYR family) (JM_20a9)	226285694	2e-20	1.13	1.31	5.39	0.98	1.6	1.64	0.55
	NADH-dehydrogenasesubunit II (JM_15b6)	68302975	2e-95	0.0009	1.85	0.07	2.02	1.08	5.4	11.3
	*NADH-dehydrogenase subunit IV (JM_129g4)	68302875	0.0	0.0006	0.41	0.21	1.29	0.79	5.59	16.7
	*NADH-dehydrogenase subunit V (JM_17g1)	68302975	2e-27	0.001	1.52	0.15	2.76	0.93	2.65	9.82
	NADH-ubiquinone oxidoreductase 105 kDa subunit (JM_85h8)	226277923	9e-09	0.0001	1.42	0.86	1.58	2.1	5.42	19.4
	Alpha-ketoglutarate decarboxylase (JM_68e9)	226283222	3e-55	0.54	0.84	0.12	3.70	5.84	1.91	0.60
	Triosephosphate isomerase ‘glycolytic enzyme’ (JM_66g5)	226279847	1e-111	0.83	0.94	5.11	1.06	1.33	0.92	0.98
**2**	Y20 (JM_2a8)	17980997	Control spot	1.4	0.4	0.72	0.24	1.8	2.18	1.18
	Mannosyltransferase (JM_2h7)	14161488	Control spot	0.58	0.97	0.15	1.63	4.9	1.43	0.96
**3**	Hydrophobin	226280363	4e-22	17.9	4.5	0.46	1.2	15.1	0.22	5.3
**4**	ABC transporter ‘cdr4’ (JM_15g4)	226282148	2e-75	1.3	0.06	7.2	0.47	0.94	1.15	1.3
	Peptide transporter ‘ptr2’ (JM_67b9)	226279920	7e-85	0.43	1.30	0.06	3.40	2.61	0.41	0.44
	Transmembrane amino acid transporter protein (JM_39g8)	226282232	2e-136	1.19	1.14	6.37	0.84	1.46	0.96	1.24
	Phospholipid-translocating P- type ATPase domain-containing protein ‘flippase ’ (JM_14d8)	226279539	0.0	0.71	0.57	5.69	0.84	1.13	0.99	0.86
**5**	hsp70 (JM_94f7)	226282776	0.0	2.5	0.27	0.11	2.5	9.3	1.1	1.3
	hsp88 (JM_24c7)	226281628	2e-35	13.8	1.3	0.10	1.2	2.6	0.6	0.23

Geometric mean of linear values from microarray data; E_2_ (17β-estradiol) vs Controls (untreated control plus ethanol-treated control), M: Mycelia. 1: Energy production, 2: Yeast genes 3: Mycelial genes, 4: Transporters, 5: Heat-shock response.

*Mitochondrial encoded gene. GI: GenBank accession number from www.ncbi.nlm.nih.gov. BLASTX expect value ≤1e-04 was considered significant.

**Table 2:** Sequence analysis of E_2_ regulated gene and expression values in comparison to controls at various time-points during M-to-Y transition.

Gene product		Accession number (GI)	BLASTX (Fungi); E-value	M	E_2_:Control (Ratio)					
				Hours	Hours					
				0	4	12	24	72	144	216
**6**	Benzoate 4-monooxygenase cytochrome P450 (JM_33b9)	226283340	1e-60	1.49	1.01	6.24	1.54	1.18	0.59	1.16
	Major facilitator superfamily/benzoate transport domain (JM_101a4)	226280741	4e-07	3.53	0.57	4.25	1.40	0.35	1.15	0.69
	Iron/manganese superoxide dismutases (JM_135a9)	226285384	2e-77	0.78	0.71	9.20	0.99	1.28	1.43	0.44
**7**	Ribosomal protein L33 (JM_99e12)	226292079	3e-21	0.71	1.20	6.31	1.17	0.66	0.80	0.77
	50S ribosomal protein L6 (JM_123f9)	226282860	2e-94	1.32	0.72	5.79	1.25	2.06	0.59	0.97
	Ribosomal protein P2 (JM_50b8)	226278075	5e-17	7.45	1.37	1.15	0.39	7.60	1.06	1.25
**8**	WD40 repeat-containing protein (JM_78e7)	226280913	6e-24	1.52	4.17	0.53	1.71	1.33	0.51	0.96
	E3 ubiquitin-protein ligase ‘ubr11’ (JM_100g9)	226282645	1e-08	0.62	1.64	4.56	0.85	1.38	1.28	1.02
	Ubiquitin ‘ubi’(JM_2f7)	25128744	Control spot	0.46	0.68	0.09	1.4	10.0	1.41	0.58
	Ubiquitin C-terminal hydrolase family protein (JM_41b1)	226280559	0.0	1.44	1.14	5.56	1.04	2.58	0.75	0.72
**9**	Palmitoyltransferase ‘erf2’ (JM_104c12)	226279299	0.0	1.74	0.07	0.46	0.66	0.28	2.63	0.97
	Small GTPase RhoA (JM_74e7)	226281788	1e-20	3.42	1.00	0.08	0.66	1.51	0.61	0.91
	Phosphatidylinositol 4-kinase ‘LSB6 serine/threonine/tyrosine protein kinases’ (JM_109f6)	226280727	1e-118	0.98	2.12	0.10	1.09	0.59	2.12	0.84
	Protein kinases (serine/threonine) ‘sgk2’ (JM_13e12)	226283417	3e-19	0.02	0.07	0.18	0.38	0.56	4.80	1.90
	Arrestin domain-containing protein (JM_41c10)	226285401	3e-73	1.52	1.85	5.28	0.31	0.33	1.75	0.63
**10**	K homology RNA-binding domain type I (JM_117f5)	22628510	3e-26	2.08	4.00	0.28	1.04	0.89	1.25	0.70
	RNA interference and gene silencing protein (JM_16e9)	226286102	6e-74	1.11	0.96	4.29	0.79	5.49	1.07	0.73
	TIA1 cytotoxic granule-associated RNA binding protein (JM_109a4)	226285398	6e-53	1.53	0.67	4.28	1.35	0.58	1.25	1.06
	Pre-mRNA-splicing factor ‘rse1’ (JM_14c5)	226278021	4e-122	0.0006	0.84	0.71	3.18	2.09	6.08	20.6
**11**	Chromodomain-helicase-DNA- binding protein (JM_36f5)	226280983	1e-157	1.12	1.19	4.21	1.23	0.73	1.44	0.41
	Acetyltransferase superfamily (JM_56g7)	226285124	7e-06	1.88	0.75	5.63	1.94	1.95	0.40	0.18
	Histone H2A (JM_83c7)	226283178	4e-28	1.66	2.16	0.09	1.09	0.84	0.51	1.21
	Chromosome segregation ATPase (JM_6g9)	226285862	0.0	0.84	1.03	1.76	0.13	0.55	1.73	1.16
**12**	Membrane-associating Marvel domain (JM_85d6)	225678699	1e-06	0.0551	2.36	0.02	6.48	0.64	0.79	0.05
	PP loop ATPase superfamily protein (JM_10b5)	226280392	2e-176	0.0004	1.14	0.43	1.57	0.89	15.2	20.1

Geometric mean of linear values from microarray data; E_2_ (17β-estradiol) vs Controls (untreated control plus ethanol-treated control), M: Mycelia. 6: Oxidative stress, 7: Ribosomal proteins, 8: Ubiquitin-mediated protein degradation, 9: Signal transduction, 10: RNA processing, 11: Chromatin structure, 12: Others. GI: GenBank accession number from www.ncbi.nlm.nih.gov. BLASTX expect value ≤1e-04 was considered significant.

### Quantitative real-time PCR expression analysis of selected genes

Confirmation of gene expression analysis of selected genes was carried out by using quantitative real-time PCR (qRT-PCR). We chose to examine some genes on the basis of their known expression patterns during form-transition to follow morphogenesis and confirm that the control cultures were progressing through morphogenesis as expected. Other genes were chosen on the basis of significant changes shown on the microarrays, or because of their potential to be involved in E_2_-mediated changes in transcription. The genes chosen for the qRT-PCR studies included those encoding heat-shock proteins (i.e., *hsp90*, *hsp70*, *hsp40*), hydrophobin, flavodoxin (*Y20*), chitin synthase (*chs4*), glucosamine-fructose-6-phospho aminotransferase (*gfa1*), β-1,3-glucan synthase (*gls1*), α-1,3-glucan synthase (*mok11*), GTPase RhoA, phosphatidylinositol 4-kinase, ATP binding cassette-cdr4 (*cdr4*), β-tubulin, ribosomal protein-l35 (*l35*), E3 ubiquitin-protein ligase (*ubr11*) and a retrotransposable element (retropepsin). All were examined during the temporal event of M-to-Y transition. Not all genes chosen for the qRT-PCR studies are known to be on the microarray, e.g., *mok11* and *hsp90*. Hydrophobin, *mok11*, *gfa1*, *chs4*, *gls1* and *Y20* were selected for qRT-PCR because of their known expression patterns, and their involvement in the structure and maintenance of the cell wall [8], [38]. Heat-shock proteins (i.e., *hsp90*, *hsp70* and *hsp40*) are well studied [39]–[41]. *cdr4* and *hsp90* were selected because *cdr1*, *cdr2* and *hsp90* in *Candida albicans* have been shown to respond to E_2_ [42]. Phosphatidylinositol 4-kinase and GTPase RhoA were chosen because they are involved in signal transduction [9]. β-tubulin and *l35* were chosen as control genes and have been used previously [8]. Details of the primers used for each of these genes are listed in Table 3.

**Table 3:** List of primers of genes selected for qRT-PCR.

Gene name	Sequence 5′ to 3′	GI no.
*hsp70*	F:GAAGGCCAGCAAGGGTATCAA	295673716
	R:TTCACCCTCATACACTTGGAT	
*hsp90*	F:CCAATTCGGCTGGTCCGCGA	295663680
	R:CGGTCGTTCTCGCCATCCGC	
*hsp40/DnaJ*	F:TCCTGCGCCTGGATTCTGTC	295672034
	R:TGAGCCTGTTGGACCGCACT	
Hydrophobin	F:ATCGTCGCCGTTGTCGCTCT	295674335
	R:ACTTGAGTACCGCTGCCATT	
GTPase RhoA	F:TGGCCGACGTTGAGGTCGAT	295659556
	R:ACCTCCTCACCCTGTTCGGT	
Phosphatidylinositol 4-kinase	F:GGACTGGACAATTGGATGAT	295675063
	R:ACTTCGCCAAGCATCAGGAT	
α-1,3-glucan synthase (*mok11*)	F:GCAGCCCAAGCTCTTGGCTCC	295668405
	R:AAATCCGGCACATATAGCCAG	
β-1,3-glucan synthase (*gls1*)	F:TGAGGCCTAGCCGTCAAATCCG	295664766
	R:ACCCCGTAATGCCAACCCCG	
Flavodoxin (*Y20*)	F:CTTGAGTAAGGACGAAGAATCGC	295663610
	R:GCCAAGGGCGTTTAGGGGGC	
Retropepsin	F:AACCTCCCAGATGCCCTGCCA	295670764
	R:TGGGCCAGCCAGAGGTCAT	
*cdr4*	F:CGGGTGAAGGTCTAAACGTT	295672460
	R:GGATCCAGGGGCTGCACCAAT	
β-tubulin	F:TGGCCACTTTCTCTGTCGTTC	295667874
	R:CAGGGTGGCATTGTATGGCT	
Ribosomal protein (*l35*)	F:GAAATACCTACCCCTCGAC	295669730
	R:CTTCTTCTGCCTCTCTGTCT	
E3 ubiquitin-protein ligase (*ubr11*)	F:GCAGAGAGCAGCAACAGTAT	295673454
	R:TTCCACCGCATTCAGCAACGT	
Glucosamine fructose-6-phosphate amino-transferase (*gfa1*)	F:AAGCCGTCATGTCTGGCGAG	295672889
	R:GATGCGCTCCGTTTCAGATG	
Chitin synthase	F:AGCGAGCGCAGAAAATCGAA	157285015
	R:GAGCGTGGGAACATGCACGA	

**Figure 3:**
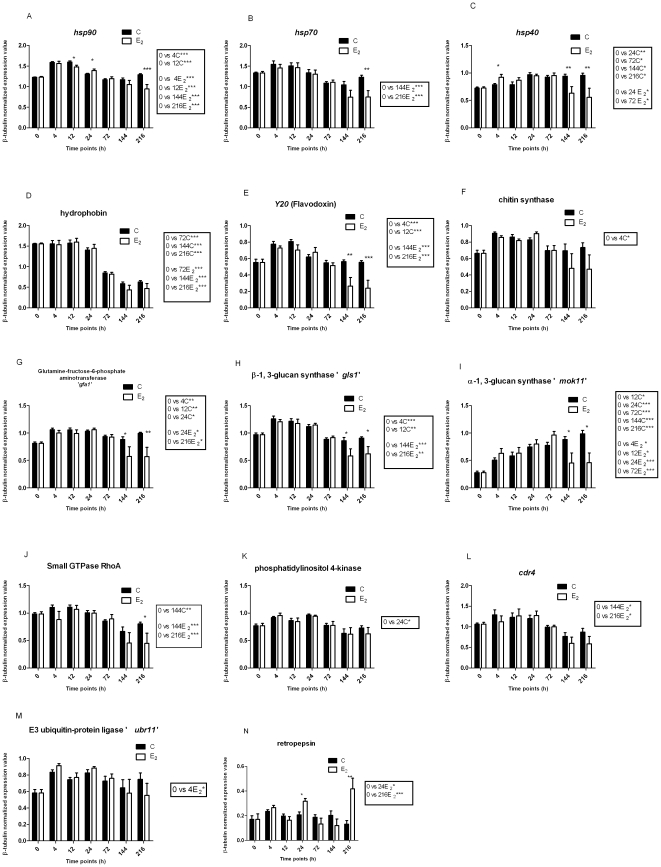
Quantification of expression of genes by controls and E_2_-treated Pb01 cells during M-to-Y transition. Panel A illustrates *hsp90*, B; *hsp70*, C; *hsp40*, D; hydrophobin, E; *Y20*, F; chitin synthase, G; *gfa1*, H; *gls1*, I; *mok11*, J; GTPase RhoA, K; phosphatidylinositol 4-kinase, L; *cdr4*, M; *ubr11*, and N; retropepsin. The expression value of each gene was obtained from β-tubulin-normalized quantitative real-time PCR (qRT-PCR), plotting controls (C; untreated plus ethanol-treated control) vs E_2_-treated at different time points. The measured quantity of each Pb01 gene from each sample was normalized by using C_T_ values obtained for the β-tubulin qRT-PCR on the same plate. The relative quantification of each gene and β-tubulin gene expression was determined by a standard curve (i.e. C_T_ values plotted against logarithm of the DNA copy number). The values represent the number of copies of the cDNA of each gene divided by the number of copies of the cDNA of the β-tubulin gene. The data represent the mean (±SEM) of triplicate of qRT-PCR runs from RNA samples of two independent biological replicates used in the microarray hybridization. Statistical comparison between C (controls) and E_2_ (E_2_-treated) samples was done at each time point and significant differences denoted on the figure above the bars by one or more asterisks, which indicate the p value (* p<0.05, ** p<0.01, *** p<0.001). The box on the right side of each figure shows the result of statistical comparison between C (controls) or E_2_ (E_2_-treated) samples at various time points and the 0 h baseline time point.

Figures 3A-N show β-tubulin normalized relative mRNA levels of each gene determined by qPCR; data from *l35* and β-tubulin controls are not included (data not shown). The correlation coefficient of qRT-PCR results and microarray data was positive for 11 of the 14 functional category genes examined. Different normalization steps used for qRT-PCR and microarray likely resulted in discrepancies in the correlation of expression determined by microarray versus that found by qRT-PCR.

#### Heat-shock response

Previous studies with *Paracoccidioides* have shown that *hsp90* or *hsp70* are induced during M-to-Y transition and are crucial for its adaptation to increased temperature [11], [40], [43]. In the present study, *hsp90*, *hsp70* and *hsp40* showed differentially regulated transcription by qRT-PCR in response to E_2_. *hsp90* and *hsp70* showed low numbers of transcripts at initial time points (4 or 12 h) during transition in response to E_2_ (Figures 3A and 3B). In contrast, *hsp40/DnaJ* showed high transcript numbers at the initial time points (4 and 12 h) in response to E_2_ (Figure 3C). However, we observed a significant drop in transcript numbers for *hsp90*, *hsp70* and *hsp40* at later time points (i.e., 144 or 216 h) in E_2_-treated samples in comparison to controls (Figure 3A–C); a significant drop occurred for only for *hsp70* or *hsp90* in comparison with the baseline time point (i.e., 0 h) in E_2_-treated samples (Figure 3A and 3B).

#### E_2_ affects cell wall and membrane-related genes during transition

Dimorphic transition (i.e., M-to-Y) causes changes in the composition of cell wall components and plasma membrane lipids [6]. Therefore, to understand how the presence of E_2_ affects transcription of genes related to the M-to-Y morphogenesis of Pb01, we performed qRT-PCR for M- or Y-form related or specific genes.

The expression of the M-form related gene, hydrophobin, was maintained in control and E_2_-treated samples until 72 h compared to the baseline 0 h time point (Figure 3D). However, somewhat higher mRNA expression was seen for hydrophobin in E_2_-treated samples in comparison to control during the initial 24 h of transition; at later times (144 and 216 h) a significant drop in expression was observed in both control and E_2_-treated samples (Figure 3D). As anticipated, in comparison with the baseline 0 h time point, significant upregulation of transcripts of the flavodoxin-*Y20* (Y-related gene), chitin synthase, glucosamine-fructose-6-phospho aminotransferase and β-1,3-glucan synthase occurred at 4 or 12 h in controls compared to E_2_-treated samples (Figures 3E–H). Thus, control cultures behaved as expected, with decline of transcripts of an M-related gene and increased transcription of genes associated with M-to-Y transition and the Y form.

The primary polysaccharide synthase of the yeast cell wall, α-1,3-glucan synthase (Y-related gene), showed significant upregulation of transcripts in control and E_2_-treated cells until the 72 h time point compared to the 0 h baseline time point, but showed significantly reduced transcript expression in E_2_-treated samples at later time points (144 and 216 h)(Figure 3I). The increased mRNA expression α-1,3-glucan synthase in the control cultures indicated a change to the Y-form that was corroborated by the observed morphology (Figure 1A). In contrast, a significant decrease in mRNA expression of α-1,3-glucan synthase was observed for E_2_-treated cells from 72 h onwards, which correlated with the observed lack of significant morphological changes in those cultures.

### Signaling genes potentially involved in M-to-Y transition

Genes such as palmitoyltransferase ‘erf2’, small GTPase RhoA, arrestin domain-containing protein, phosphatidylinositol 4-kinase, serine/threonine-protein kinase are related to G-protein mediated signaling and the phosphatidylinositol signaling system. By microarray all but the arrestin domain-containing protein showed lower transcript expression in E_2_-treated cultures at the initial time points (4 or 12 h) compared to controls (Figure 2A). In contrast, the gene encoding for an arrestin domain-containing protein showed higher transcript expression on the microarray (Figure 2A).

qRT-PCR data for GTPase RhoA (Figure 3J) and phosphatidylinositol 4-kinase (Figure 3K) showed a trend similar to that of the array data (Figure 2A), where GTPase RhoA showed fewer transcripts at the 4 or 12 h time points, and phosphatidylinositol 4-kinase showed lower mRNA expression at the 12 or 24 h time points in E_2_-treated samples compared to controls (Figure 3J and 3K). qRT-PCR also showed a significant increase in phosphatidylinositol 4-kinase transcript numbers at 24 hours of transition in controls compared to E_2_-treated samples (Figure 3K). We observed a significant drop in mRNA expression of GTPase RhoA by qRT-PCR in E_2_-treated samples compared to controls or to the baseline time point 0 h at a later time point (i.e., 216 h). This gene also showed low level of mRNA expression at the 4 h time point in response to E_2_ treatment (Figure 3J). Thus, these signaling genes likely have a critical role in dimorphic switch from M-to-Y.

### Transporter and detoxification genes

The microarray showed that mRNA for several genes were highly expressed in response to E_2_ at the 12 h time point (Figure 2A). These included benzoate 4-monooxygenase cytochrome P450, which may be involved with hydroxylation of E_2_ [44], major facilitator super family (MFS)/benzoate transport, and iron/manganese superoxide dismutases. In contrast, the stress responsive gene, *cdr4*, which encodes multidrug transporters of the ABC family, showed low mRNA expression in E_2_-treated samples at 4 h followed by a large increase in mRNA expression at 12 h compared to controls (Figure 2A). qRT-PCR data for *cdr4* (Figure 3L) showed a trend similar to that of the microarray data. However, a significant drop in numbers of transcripts of *cdr4* by qRT-PCR was observed in E_2_-treated samples at later time points (i.e., 144 and 216 h) in relation to the 0 h baseline.

### Ubiquitin-mediated protein degradation

The gene encoding WD40 repeat-containing protein, E3 ubiquitin-protein ligase ‘*ubr11*’ and ubiquitin C-terminal hydrolase family proteins are involved in ubiquitin-mediated protein degradation and mRNA transcripts from these genes were highly expressed at 12 h in E_2_ treated samples compared to controls in the microarray (Figure 2A). qRT-PCR data for *ubr11* showed a significant increase in the number of transcripts in the E_2_-treated sample at 4 h compared to controls and consistently abundant transcripts in E_2_-treated samples until 72 h of M-to-Y transition (Figure 3M), a trend similar to that of the microarray data.

### Genes involved in energy metabolism

Thirteen clones (mitochondrial or nuclear genes) related to energy production showed altered transcriptional profiles in response to the presence of E_2_ by microarray analysis (Figure 2A). NADH-dehydrogenase subunit II, cytochrome oxidase subunit II and ATP synthase F0 subunit-9, enzymes of complexes I, IV and V of oxidative phosphorylation, respectively, showed low mRNA expression at 4 or 12 h time points during M-to-Y transition in E_2_-treated samples, but at later time points (i.e., 72 h) showed higher mRNA expression, as did other subunits of complexes I and IV (Figure 2A). The gene encoding for isocitrate lyase, a key enzyme of the glyoxylate cycle, showed low mRNA expression at the 12 h time point in E_2_-treated samples, but later time points showed higher mRNA expression (Figure 2A). These results are suggestive that due to morphological block or delay in E_2_-treated samples, glyoxylate cycle or oxidative phosphorylation was under-utilized for ATP production during the initial stage (4 or 12 h) of M-to-Y transition. We also found the gene for α-ketoglutarate decarboxylase (Figure 2A) to have high mRNA expression at later time points (i.e., 72 h) in E_2_-treated samples during M-to-Y transition. Interestingly, the gene for triosephosphate isomerase showed high mRNA expression at the 12 h time point in E_2_-treated samples (Figure 2A). This isomerase enzyme interconverts dihydroxyacetone phosphate with glyceraldehyde-3-phosphate, which proceeds further into glycolysis.

### E_2_ regulates a retrotransposon element during M to Y transition

**Figure 4:**
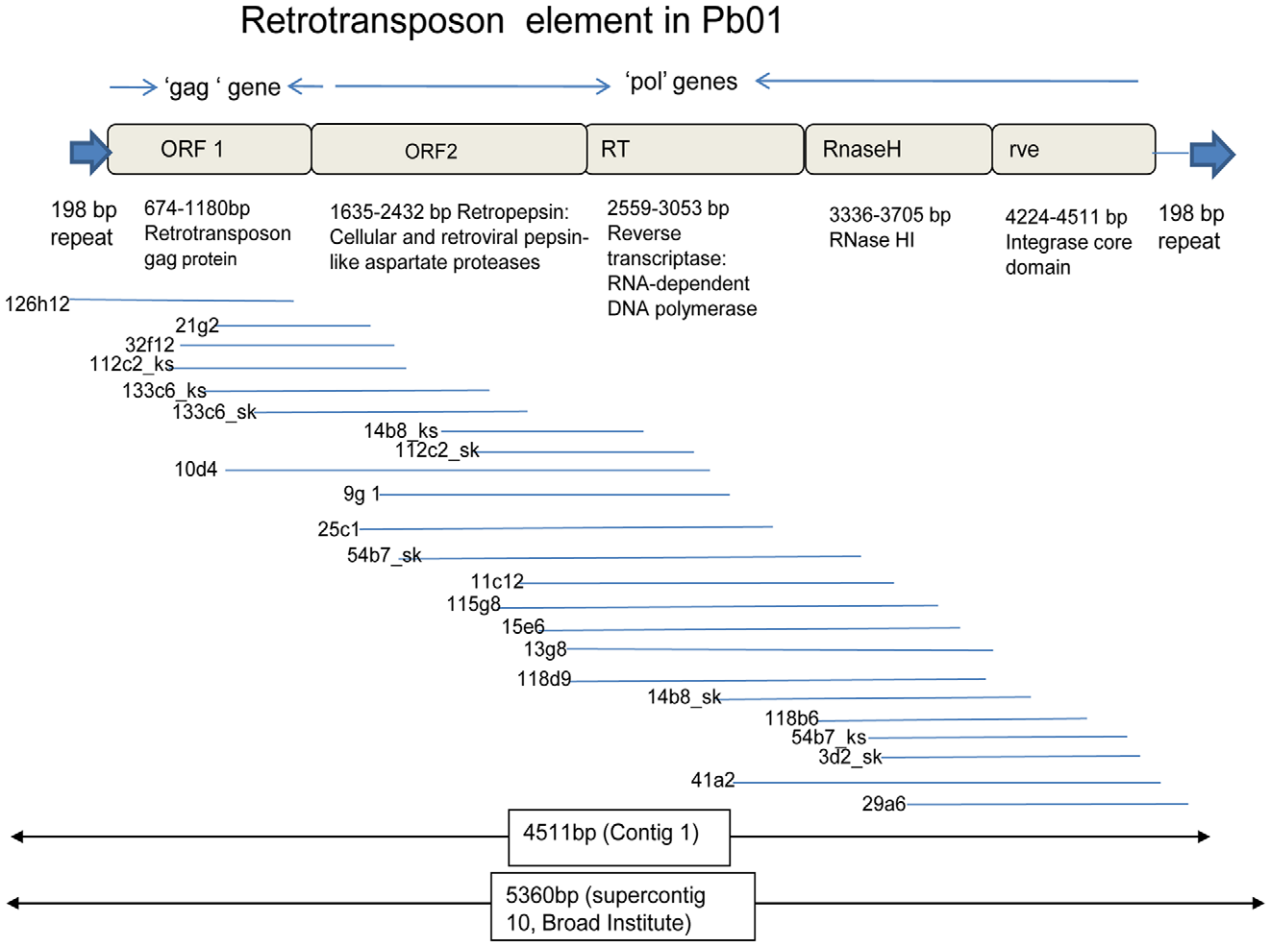
Alignment of 19 retrotransposable sequences found in contig 1 that are differentially regulated in E_2_-treated samples in comparison to control. Sequence domains were identified from conserved hypothetical protein (ORF1: Retrotransposon gag protein) of *Penicillium marneffei* (GI: 212525972), ORF2: Retrovirus polyprotein, putative, of *P. marneffei* (GI: 212525970) and hypothetical protein of *A. nidulans* (GI: 67524427).

After sequencing of differentially expressed elements on the microarray, we found 31 clones that had sequence similarity to a retrotransposon element. A lower level of mRNA expression was found at several time points until 72 h of transition in E_2_-treated cells. Sequence alignment of these clones resulted in 4 contigs and 4 singlets. The clones within each contig showed a similar trend in mRNA expression, (Figure 2B). A BLASTX search using Contig 1 (4511bp size) showed sequence similarity with a hypothetical protein of *Aspergillus nidulans* (GI: 67524427) or Pol of *Alternaria alternata* (GI: 6683624). Sequence domains were identified from a conserved hypothetical protein: ORF1, a retrotransposon gag protein of *Penicillium marneffei* (GI: 212525972), and ORF2, a putative retrovirus polyprotein of *P. marneffei* (GI: 212525970). Based on domain attribution of ORF2 in contig 1, a *pol* gene containing domains for retropepsin (PR), reverse transcriptase (RT), RNase H (RH) (Ty3/Gypsy family of RNase HI) and integrase (IN) were found (Figure 4). These were arranged in a Ty3/gypsy group order of PR, RT, RH, and IN [45]. BLASTX search of Pb01 sequences resulted in similarity with conserved hypothetical and predicted protein (i.e., GIs: 226283053, 226277605, 226286485, 226284993, 226283163 & 226279429) and with a conserved domain of cellular and retroviral pepsin-like aspartate protease (i.e., GI: 226284062) (Table 4).

**Table 4:** Analysis of the sequenced clones showing sequence similarity with retrotransposon elements.

Contigs/singlets	Size (bp)	Pb01 (BLASTX)		Other fungi (BLASTX) (taxid: 4511)	
		GI	E-value	GI	E-value
**Contig 1**	4511	Hypothetical protein (GI: 226283053)	3e-46	Pol *A. alternate* (GI: 6683624)	0.0
		Hypothetical protein (Retropepsin) (GI: 226284062)	4e-45	Hypothetical protein *A. nidulans* (GI: 67538144)	0.0
		Hypothetical protein (GI: 226277605)	5e-35	Retrotransposon gag protein *P. marneffei* (GI: 212525972)	3e-14
		Hypothetical protein (GI: 226286485)	7e-31	Retrovirus polyprotein *P. marneffei* (GI: 212525970)	0.0
		Hypothetical protein (GI: 226284993)	7e-24	Retrotransposon polyprotein *T. stipitatus* (GI: 242760779)	1e-95
		Hypothetical protein (GI: 226283163)	2e-19		
		Hypothetical protein (GI: 226279429)	8e-15		
		Hypothetical protein (GI: 226283120	3e-07		
**Contig 2**	1548	Hypothetical protein (GI: 226284593)	1e-07	Endonuclease/reverse transcriptase/Non-LTR retrotransposon*T. stipitatus* (GI: 242825272)	4e-93
**Contig 3**	2132	Conserved hypothetical protein (GI: 226283120)	7e-09	Hypothetical protein (Integrase core domain) *A. nidulans* (GI: 67524427)	2e-30
		Conserved hypothetical protein (GI: 226280246)	8e-04	Retrovirus polyprotein, (Integrase core domain) *P. marneffei* (GI: 212530907)	1e-28
**Contig 4**	1335	Hypothetical protein (Retropepsin) (GI: 226277726)	4e-100	Retrovirus polyprotein (Retropepsin) *T. stipitatus* (GI: 242775227)	6e-14
**Singlet 1**	1629	Hypothetical protein (Reverse transcriptase/Ribonuclease H, Ty1/Copia family of RNase HI in long-term repeat retroelements) (GI: 226285610)	9e-27	Hypothetical protein (Reverse transcriptase & RNase HI) *A. capsulatus* (GI:154280503)	4e-59
		Hypothetical protein (RNase H) (GI: 226280117)	2e-12		
		Hypothetical protein (Integrase core domain) (GI: 226284491)	9e-06		
**Singlet 2**	1256	No significant match		Reverse transcriptase *P. chrysogenum* (GI: 255935443)	1e-22
**Singlet 3**	859	Hypothetical protein (GI: 226285011)	2e-05	Retrovirus polyprotein (Reverse transcriptase) *T. stipitatus* (GI:242810994)	7e-04
**Singlet 4**	1168	No significant match		Retrotransposon polyprotein (Reverse transcriptase) *T. stipitatus* (GI:242805420)	2e-17
				Retrovirus polyprotein *P. marneffei* (GI: 212545550)	4e-16

Sequenced clones showing sequence similarities with retrotransposon elements are subjected to Sequencher software that resulted in 4 contigs and 4 singlets. GI: GenBank accession number from www.ncbi.nlm.nih.gov. BLASTX expect value ≤1e-04 was considered significant.

Contig 2 showed sequence homology to the endonuclease/reverse transcriptase domain of elements from *Talaromyces stipitatus* (GI: 242825272) belonging to a category of Non-LTR (long terminal repeat) retrotransposons (Table 4). Contigs 3 and 4 showed sequence homology to the integrase core domain of an element from *A. nidulans* (GI: 67538144) and to retropepsin or pepsin-like aspartate proteases from *T. stipitatus* (GI: 242775227). A BLASTX search and comparison of contig 4 with Pb01 sequences also showed homology to retropepsin or pepsin-like aspartate proteases (GI: 226277726). Amino acid sequence comparison of contig 1 (GI: 226284062) with contig 4 (GI: 226277726) resulted in no homology (E-values = 1.8). Singlets resulting from our sequence assembly showed homology to domains of RNase HI, or reverse transcriptase from other fungi (i.e., *Ajellomyces capsulatus NAm1*, *P. chrysogenum*, *T. stipitatus*, *P. marneffei*) (Table 4). qRT-PCR for the retropepsin gene from contig 1 showed minimal mRNA expression at the 12 h time and significantly higher mRNA expression at the 24 and 216 h time points in E2-treated samples (Figure 3N).

## Discussion

We describe the effect of E_2_ on the transcriptional profile of *Paracoccidioides* during M-to-Y transition. E_2_ may block or delay the morphological transition by modulating gene expression of several functional categories of genes including: heat-shock response, cell wall maintenance/remodeling, energy metabolism, transporter and detoxification, cell signaling, and retro-transposable elements. Our microarray data, though it lacks a complete coverage of the genome (i.e., we estimate the microarray covers approximately 25% of the genome [8]), provide an overview of the response of *Paracoccidioides* to E_2_ during M-to-Y transition. We considered the microarray data as leads for further evaluation of transcripts by qPCR. These results corroborate those from previous studies on temperature-induced dimorphism, as well as extend the results on E_2_-induced inhibition of M-to-Y transition.

After an increase in temperature induces transition from M-to-Y, heat shock proteins or cell wall structure remodeling genes are influenced. Our qRT-PCR data showed *hsp90* and *hsp70* were less responsive to the presence of E_2_ with the onset of transition. In *Paracoccidioides*, *hsp70* is induced during M-to-Y transition with a transient accumulation of unspliced *hsp70* mRNA transcripts [40]. However, after adaptation to 36°C yeasts acquire proficiency in splicing [40]. Heat shock proteins/genes are modulated in response to various stimuli, including temperature in other fungi [46]–[48].

In our study, *hsp40/DnaJ* showed higher mRNA expression at initial time points in response to E_2_. hsp40 directly interacts with hsp70 and hsp90 as a co-chaperone protein [49], [50]. The members of this family of DnaJ/hsp40 proteins are evolutionarily conserved and characterized by the presence of one or more J domains in their N-terminus; the J domain is important for the hsp40-hsp70 interaction and stimulation of ATPase activity [49], [51]. In mammals, hsp90, hsp70 and hsp40 take part in the estrogen hormone-receptor hetero-complex to assist proper protein folding and activation by hormone binding [52]. In addition, in yeast, Ydj1/hsp40 mutants displayed high basal levels of steroid receptor activity in the absence of estrogen suggesting that Ydj1/hsp40 is important for the receptor regulation by the hsp90 folding pathway [53]. Upregulation of *hsp40* and downregulation of *hsp90* at early time-points in response to E_2_ possibly delays or interferes with the normal cellular responses of *Paracoccidioides* to change in temperature, thus affecting subsequent changes of morphology.

A critical morphological difference between mycelial and yeast is the composition of the cell wall. The primary cell wall constituents of both are glucans, chitin, proteins and lipids [6]. However, yeast have a larger percentage of chitin than do the mycelia [6]. Our data showed *gfa1* and chitin synthase had lower mRNA expression in E_2_-treated samples at earlier time points. Glucosamine-fructose-6-phospho aminotransferase (i.e., *gfa1*) is essential in *S. cerevisiae*, catalyzing the first step in the hexosamine biosynthesis pathway [54]. Chitin synthase is a membrane-bound protein, involved in the polymerization of chitin in the fungal cell wall [55]. Our data suggests that E_2_ may inhibit the change in morphology of *Paracoccidioides* by modulating early mRNA expression of *gfa1* and chitin synthase.

In both M- and Y-forms, 40% of the cell wall is glucan. In the M-form, the wall consists mainly of β-glucan with β-(1,3)–glycosidic linkages, whereas the Y-form mainly contains α-glucan with α-(1,3)–glycosidic linkages; only 4–5% of the cell wall of the Y-form is β-glucan [38]. The significant drop in the mRNA expression of α- and β-glucan synthases, *gfa1*, *Y20*, *hsp70*, *hsp90* and *hsp40* at late time points in E_2_-treated samples supports the lack of morphological change into yeast. Remodeling of the cell wall and reorganization of membrane-lipid occurs as *Paracoccidioides* adapts to changes in temperature [56], [57]. Our data reflect this for the control cultures and indicate that while M-to-Y transition is complete in control cultures, it has been inhibited in E_2_-treated cultures.

In addition to changes in structural components, the two morphological forms differ in energy metabolism. Yeast cells are able to utilize aerobic or anaerobic metabolism, including the glyoxylate pathway, for their energy needs, whereas mycelial cells tend to be more aerobic [15], [16]. Our data suggest that energy needs in E_2_-treated samples have been maintained through glycolysis.

We found evidence that *Paracoccidioides* responds to E_2_ by activating genes that might remove or metabolize the hormone. In other yeasts, E_2_ enters into the cells by diffusion [58]. Once E_2_ is inside *C. albicans*, the increased mRNA expression of *cdr1* and *cdr2* likely increases production of efflux pumps, which remove E_2_ from the cells [42]. We observed increased expression of the *cdr4* gene at 12 and 24 h time points, which is suggestive that the stimulated mRNA expression in the presence of E_2_ was an attempt to use an efflux pump to remove the E_2_.

We also observed upregulation of transcripts of the major facilitator superfamily gene with a benzoate transport domain, benzoate 4-monooxygenase cytochrome P450 in E_2_-treated cultures; this protein shares significant amino sequence similarity with CYP3A4 (E = 5e-13; GI: 6470135) and CYP3A7 (E = 5e-23; GI: 30840237) in humans. Isoforms of the cytochrome CYP3A4 have strong activity in the formation of 2-hydroxyestradiol and 4-hydroxestradiol from E_2_ [44]. Induction of CYP3A4 by E_2_ may be a mechanism for detoxification of E_2_, analogous to the hydroxylation of progesterone by *Trichophyton mentagrophytes*, which reduces the inhibitory effects of progesterone on growth [59]. Possibly, *Paracoccidioides* may reduce the intracellular burden of E_2_ by hydroxylation or by an efflux mechanism, to remove the inhibitory actions of E_2_ that prevent progression of dimorphism.

We found that many genes changing in mRNA expression involved signaling pathways. We observed down-regulation of mRNA of small GTPase RhoA (G-protein) and palmitoyltransferase ‘erf2’ in E_2_-treated samples. G-protein family proteins, such as small GTPases or RAS-related proteins, appear critical to the dimorphism of *P. marneffei* by regulating cell polarity [60], or to cell wall remodeling in *Paracoccidioides* by regulating the α- or β-glucan synthases needed during M-to-Y transition [7], [61]. Gene silencing of Rho-like GTPase PbCdc42 in *P. brasiliensis* resulted in a reduced yeast cell size and fewer buds per cell [18]. Palmitoyltransferase ‘erf2’ is involved in the attachment of palmitic acid to G-proteins, and affects G-protein function by targeting it to a specific membrane domain, regulating responsiveness to upstream signaling [62]. Other genes associated with signaling, phosphatidylinositol 4-kinase and protein kinase (serine/threonine), showed low mRNA expression in E_2_-treated samples. These genes are involved in the phosphatidylinositol signaling pathway, important for the remodeling and reorganization of cell wall and cell membrane [63].

Ca^2+^, calmodulin and calcineurin have been reported involved in morphogenesis and calcium homeostasis during M-to-Y transition in *Paracoccidioides* [64], [65]. Bastos et al. [9] showed induction of Rho GTPase activating protein, UVSB phosphatidylinositol-3 kinase, myo-inositol-1-phosphate synthase and phosphatidylinositol transfer protein in a transcriptome analysis 22 h into M-to-Y transition by Pb01. Chen et al. [66] showed increased transcript numbers of adenylate cyclase, as well as a G-protein subunit (*GPB1*), with the onset of M-to-Y in *P. brasiliensis*. However, at a later time point (i.e., 240 h) decreased numbers of transcripts of *GPB1* versus increased numbers of transcripts of G-protein subunits *GPA1* and *GPG1* were observed [66], indicating modulated expression of these genes control the signal needed for M-to-Y dimorphism. We observed reduced numbers of transcripts for small GTPase RhoA, palmitoyltransferase ‘erf2’, phosphatidylinositol 4-kinase and protein kinase, all involved in signal transduction, during initial stages of M-to-Y transition. This would reduce the magnitude of signaling during the dimorphic switch in E_2_-treated samples, and further implicates the critical role of signaling regulation in dimorphism.

In the early phase of transition in E_2_-treated samples, we observed increased mRNA expression for genes of E3 ubiquitin-protein ligase ‘*ubr11*’, WD40 repeat-containing protein, arrestin domain-containing protein and ubiquitin C-terminal hydrolase. E3 ubiquitin-protein ligase is involved in the ubiquitination of the arrestin-protein complex formed with G-protein coupled receptor (GPCR), targeting the GPCR to the proteasome for proteolytic cleavage [67], effectively, terminating the signal or desensitizing GPCR signaling. It also plays a role in attenuation of cAMP signaling [67]. Others have shown elevated intracellular cAMP levels correlate with transcript expression of adenylate cyclase during M-to-Y transition by *Paracoccidioides* [66], [68]. In *Rhizopus nigricans*, a plasma membrane-bound progesterone receptor coupled to G-proteins has been reported, where progesterone inhibits fungal growth by a G-protein mediated decrease in intracellular cAMP [69].

The mRNA changes in these signaling pathways shown by our data may provide insight into the mechanism of E_2_ action. Although, the action of steroid molecules normally occurs through classic steroid receptors, it can also be mediated through alternative molecules residing in the membrane. The induced transcripts of E3 ubiquitin-protein ligase, arrestin-domain containing protein, WD40 repeat-containing protein and ubiquitin C-terminal hydrolase, and reduced transcripts of GTPase RhoA and palmitoyltransferase ‘erf2’, in E_2_-treated samples during initial phase of transition suggests that a WD40 repeat-containing protein may serve as an anchor for ubiquitination of the arrestin-protein by E3 ubiquitin-protein ligase when the arrestin is bound to GPCR or GPCR-like protein, directing them to the protein degradation pathway to terminate GPCR or GPCR-like protein mediated signaling in E_2_-samples. This suggests that E_2_ binds to a transmembrane protein that has affinity for G-proteins.

We identified several contigs or singlets, showing sequence similarity with retrotransposable elements. These elements were more highly expressed with the onset of M-to-Y transition in the controls than in the E_2_-treated samples. These data support the existence of retrotransposable elements in the genome of Pb01; transcripts of Ty-like elements have been reported in the Pb01 isolate [13]. Furthermore, we found a 198 bp long terminal repeat (LTR) similar to Ty-like elements, which include genes encoding both structural and enzymatic proteins, and remnants of retrotransposable elements. We showed mRNA expression of retrotransposable elements was modulated in the presence of E_2_ during M-to-Y transition. Although, expression of these elements may lead to deletion, insertion or translocation of genes in the genome, their role during M-to-Y transition requires further study. A genomic survey of *P. brasiliensis* strains revealed eight new Tc1/mariner families (i.e., DNA transposons), referred as to as TremA (Transposable element mariner) through TremH [70]. We found no Trem transposons in our sequence data.

Although evidence has been presented regarding how E_2_ inhibits morphological transition from mycelia to the parasitic yeast, questions remain. Confirmation that E_2_ binds to a true receptor in *Paracoccidioides* to modulate gene expression and the entire sequence of events that take place in the presence of E_2_ during M-to-Y inhibition remains to be elucidated. Nevertheless, our data provides insights into how exposure to exogenous E_2_ inhibits M-to-Y transition in *Paracoccidioides*, and how E_2_ effects could lead to the strong gender bias for the development of the clinical disease [4].

## Materials and Methods

### Pb01 strain and maintenance

We used *Paracoccidioides lutzii* strain Pb01 (ATCC MYA-826) to construct a random sheared gDNA array for evaluation of gene expression [8], similar to studies done in other laboratories [9], [13], [40]. M- and Y-forms were maintained on modified McVeigh-Morton medium (MVM) agar slants at ambient temperature and at 37°C respectively, and were transferred monthly [71].

### Pb01 growth and nucleic acid extraction

M culture filtrate was filter sterilized from MVM broth cultures (Pb01) after 2–4 weeks of growth at 25°C on a gyratory shaker at 150 r.p.m. and stored at 4°C for use as a growth supplement. M spent culture medium was used as growth supplement (2∶1 ratio of fresh liquid MVM to spent culture filtrate) as described previously for the growth of M liquid cultures [36], [72]. Two-week-old M slants were used to inoculate 300 ml of supplemented liquid MVM media, and liquid cultures were grown for 8 d on a gyratory shaker at 150 r.p.m. at 25°C. After the 8 d growth period, 17β-estradiol (E_2_; Steraloids, Newport, RI, USA) was added to M-phase cultures at a final concentration of 2.6×10^−7^ M in 0.1% absolute ethanol or ethanol alone (final concentration 0.1%) [36]. This concentration of E_2_ is a high physiological concentration and was selected to achieve a high proportion of inhibition of M-to-Y transition. The physiological and pharmacological concentrations of E_2_ relevant to human females range from 2×10^−10^ M to 2×10^−6^ M [4]. Morphological transition from M-to-Y was induced by increasing the incubation temperature from 25°C to 37°C. Samples from 8 d old M cultures were collected, before adding E_2_ or ethanol alone and served as the 0 h time point. After the temperature switch, samples were harvested from untreated controls, ethanol-treated controls, or E_2_-treated cultures at 4, 12, 24, 72, 144, or 216 h time points by centrifugation at 1500 r.p.m. for 10 minutes followed by snap-freezing of the fungal mass in liquid nitrogen. These samples were stored at −80°C until RNA extraction was performed. Liquid cultures were evaluated for contaminating organisms by plating samples on 5% sheep blood agar (BD Diagnostic Systems, MD, USA) followed by incubation for 2–3 days at 37°C, and by microscopic examination of Lactophenol cotton blue (Becton, Dickinson and Company, Mexico) stained slides of Pb01; the slides were also used for morphological evaluation and confirmation of M-to-Y transition.

#### RNA extraction

Fungal cells were thawed in TRIzol reagent (Invitrogen, Carlsbad, CA) followed by cell disruption with 0.5 mm zirconia/silica beads (Biospec) using a BeadBeater apparatus (Biospec Products, Inc., Bartlesville, OK) at high speed setting (3 pulses of 12–15 sec). Total RNA was extracted using TRIzol reagent per the manufacturer's instructions. Contaminating gDNA was removed per the manufacturer's instructions using an RNeasy mini spin column (RNeasy mini kit, Qiagen Science, MD, USA) and an RNase-free DNase set (50) (Qiagen, GmbH, Hilden). The quantity and quality of the extracted RNA were assessed by A_260 nm_/A_280 nm_ ratio as well as by electrophoresis through a 1.5% agarose gel stained with ethidium bromide for the presence of intact 18S and 28S ribosomal RNA bands, visualized by UV transillumination at 302 nm.

#### DNA extraction

Genomic DNA (gDNA) was extracted from yeasts collected from 5-day-old liquid cultures (37°C, shaking at 150 r.p.m.) using DNeasy Plant Mini Kit (Qiagen, GmbH, Hilden) per the manufacturer's instruction. The quantity and quality of the extracted gDNA was assessed by A_260 nm_/A_280 nm_ ratio and electrophoresis through a 0.8% agarose gel stained with ethidium bromide and visualized by UV transillumination.

### cDNA labeling, hybridization procedure and image acquisition

Total RNA (48 µg) was used for cDNA synthesis using anchored oligo-dT (5′ TTT TTT TTT TTT TTT TTT TTV N 3′). Fluorescently labeled cDNA was made by incorporating Cy5 or Cy3 labeled dUTP during reverse transcription of poly-adenylated (PolyA) mRNA. An equal amount of total RNA from untreated controls from each time-point was pooled to generate a Cy5-labeled cDNA reference sample. Samples from each time point were individually labeled with Cy3.

Cy3-labeled samples were competitively hybridized against the Cy5-labeled reference sample on a pre-hybridized microarray slide (random shear gDNA microarray [8]). cDNA labeling and pre-hybridization procedures were followed as previously described [8]. Samples of labeled cDNA for hybridization were concentrated using a Microcon® YM-30 Centrifugal Filter Device (Millipore) and suspended to 40 µl with 3× saline-sodium citrate buffer (SSC; Sigma-Aldrich, St. Louis, MO), 0.1% sodium dodecyl sulfate (SDS; Sigma-Aldrich). These samples were preincubated at 100°C for 2 minutes, 37°C for 15–25 minutes, transferred onto a microarray slide, covered with a cover glass and placed in hybridization chambers. Hybridization was carried out at 50°C for 14 to 18 hours followed by washing, in order, with 2× SSC with 0.03%SDS, 2× SSC, 1× SSC and 0.2× SSC for 2–5 minutes in each solution. Slides were dried by centrifugation and scanned within 24 hours. Scanner settings used 10 µm pixel size and photomultiplier tube gains for both detection channels, 635 nm (Cy5-red color) and 532 nm (Cy3-green color) were 540 nm and 500 nm, respectively (GenePix® 4000B Microarray Scanner, Axon Instruments, Union City, CA). All hybridization experiments were done in duplicate using samples from each of two independent cultures.

### Microarray data analysis

Microarray hybridizations were imaged using a GenePix scanner. Images were processed using GenePix Pro 6.1.0.4 software. GenePix files were imported into R/Bioconductor [73], using the limma package [74], removing spots having quality flags of −50 or less. Analysis of array backgrounds revealed evidence of spatial gradients, so background correction was applied to each array using the normexp method with an offset of 50 [75]. We applied the arrayQualityMetrics package [76]. We normalized between arrays using variance stabilization from the vsn package [77], allowing for 20 percent outliers. We then normalized within arrays using loess to remove non-linearity from the MA curves (M and A are defined as: M = log_2_ (I1)−log_2_ (I2), A = 1/2 (log_2_ (I1)+log_2_ (I2)), where I1 and I2 are the intensities of the two channels). Finally, we obtained standardized log_2_ ratios by dividing the log_2_ ratios of each array by its median absolute deviation. Initially, we tried to analyze the data using linear modeling in the limma package, but found that many of the top results were due to outliers in which only one of the replicate samples had an extremely high value. Therefore, we performed the analysis using a non-parametric rank product methodology [78], which is robust to outliers. For each time point we compared the E_2_-treated samples with pooled untreated control and ethanol-treated control samples, performing the rank product test for 1000 permutations. The controls were pooled to minimize or eliminate any effects that the vehicle for the E_2_, ethanol, might have on the analysis on a gene-by-gene basis. Genes that had a vehicle effect should show increased variability of expression in the pooled control samples and therefore lower the resulting t statistic. Pooling also increased the accuracy of our analysis by increasing the number of available control samples. We identified up- and down-regulated genes on the basis of estimated p-value with a cutoff of 1e-3 in the rank product test for all time points.

### Sequencing and bioinformatics analysis

Differentially expressed genes were manually inspected to exclude outliers in the controls (pooled untreated control and ethanol-treated control). Ninety-two array elements with a distinguishing expression pattern between E_2_-treated samples and controls were selected for sequencing. For each clone, 5 µl of phage from the cloned stock was used as a template for PCR using Platinum® PCR SuperMix as per manufacturer's instruction (Invitrogen) as described previously [8]. Amplified products were purified using QIAquick PCR purification kit as per manufacturer's instruction (Qiagen, MD, USA). DNA sequencing reactions were performed from both forward and reverse primers using ABI Big Dye v3.1 sequencing chemistry at the Protein and Nucleic Acid facility at Beckman Centre, Stanford University. Each sequence was processed and aligned to generate contigs or singlets using the DNA analysis software Sequencher™ v4.8 (Gene Codes Corporation, MI, USA). The BLASTX program [79] with a cutoff expected value of ≤1e-04 at NCBI (http://blast.ncbi.nlm.nih.gov/Blast.cgi) was used as a match to assign gene and functions to the sequenced DNA clones.

### Quantitative real-time PCR

Real-time PCR reactions were performed in a 384-well format using Applied Biosystem 7900HT Fast Real-Time PCR system (7900HT Fast System). *POWER* SYBER Green RNA-to-C_T_ 1-step kits (Applied Biosystem, Foster City, CA, USA) were used per the manufacturer's instructions in all real-time PCR reactions. Each PCR reaction was performed in 20 µl total volume, with 100 ng of total RNA as a template. The thermo-cycling conditions were comprised of an initial step at 48°C for 2 min for reverse transcription, followed by 95°C for 10 min, and 40 cycles of 95°C (denature) for 15 s, 60°C (anneal/extension) for 1 min. A dissociation curve was performed for primer specificity. All reactions were done in triplicate. Negative control reactions included no template or no reverse transcriptase to check undesirable amplifications.

We used the β-tubulin gene from gDNA dilutions ranging from 3 to 3×10^5^ genome copies in 10-fold increments to generate a standard curve to determine copy numbers, with a single gene copy per genome [80], [81]. A linear relationship was obtained by plotting the threshold cycle against the logarithm of known amount of initial template [82]. Accordingly, from our previous study and other gene expression studies on Pb01 [8], [40], the β-tubulin gene was used as the normalizer. Primers were designed from an exon-exon boundary or with a gap of intron sequence or from the 3′ exonic sequence; primer sequences are described in Table 3. Real-time PCR reactions were performed on RNA samples from two independent biological replicates, the same as used in the microarray hybridizations.

### Statistical analysis

The significance of the difference of the mean values of the data in independent samples at given time-points in real-time PCR figures [e.g. at 4 h comparing control and E_2_, (4C vs 4E_2_)] was established by two-sample unpaired t-tests using GraphPad Software (GraphPad Prism v 5.0, GraphPad Software). To compare control or E_2_-treated samples, at all time points to the baseline time point (0 h) in real-time PCR data, one-way ANOVA with a Dunnett post-test was performed. Comparisons of the controls (i.e., untreated and ethanol treated control) in the microarray and qRT-PCR data were also carried out and showed significant differences in gene expression (p≤0.05) at single time points for few genes in the microarray data (e.g., membrane-associating marvel domain, major facilitator superfamily, and retropepsin), as well as for the qRT-PCR data set where β-1,3-glucan synthase, α-1,3-glucan synthase, and *hsp90* showed a difference between the controls. However, comparison of individual controls with E_2_-treated showed the same trend, either up- or down-regulation of mRNA expression, as the comparison of expression in the presence of E_2_ with the overall expression in the pooled controls; data are presented based on the pooled controls. Although, the ethanol used as the vehicle control had some effect on several genes at a single time point, it had little or minimal effect on subsequent comparisons made when pooling the controls. Thus, we pooled the untreated and ethanol controls to increase the number of replicate control samples and to reduce false positive results due to the vehicle ethanol. To determine correlation between microarray and real-time PCR data, log_10_ transformed β-tubulin normalized relative expression value of a gene from controls or E_2_-treated samples from microarray was plotted against log_10_ value from real-time PCR at 0, 4, 12, 24, 72, 144 and 216 h time-points. Spearman and Pearson's correlation coefficients were determined in GraphPad Software for the entire temporal timeframe. A p-value<0.05 was considered significant in all analysis.

### Accession numbers

The GenBank accession numbers (dbGSS id) for DNA sequences are available under F1778766-F1779357 and JM175074-JM175450. The microarray data discussed in this publication have been deposited in MIAME compliant database - NCBI's Gene Expression Omnibus [83] - and are accessible through GEO Series accession number GSE30466 (http://www.ncbi.nlm.nih.gov/geo/query/acc.cgi?acc=GSE30466).
